# Methylene Blue Mitigates Acute Neuroinflammation after Spinal Cord Injury through Inhibiting NLRP3 Inflammasome Activation in Microglia

**DOI:** 10.3389/fncel.2017.00391

**Published:** 2017-12-11

**Authors:** Zhi-Hang Lin, Si-Yuan Wang, Li-Li Chen, Jia-Yuan Zhuang, Qing-Feng Ke, Dan-Rui Xiao, Wen-Ping Lin

**Affiliations:** ^1^Department of Pharmacy, Affiliated Quanzhou First Hospital of Fujian Medical University, Quanzhou, China; ^2^Department of Orthopedic Surgery, The Second Affiliated Hospital, Fujian Medical University, Quanzhou, China; ^3^Nursing Department, Fujian Medical University Affiliated Provincial Clinical Medical Institute, Fuzhou, China; ^4^The School of Nursing, Fujian Medical University, Fuzhou, China

**Keywords:** spinal cord injury, methylene blue, NLRP3, locomotor function, neuroinflammation

## Abstract

The spinal cord injury (SCI) is a detrimental neurological disease involving the primary mechanical injury and secondary inflammatory damage. Curtailing the detrimental neuroinflammation would be beneficial for spinal cord function recovery. Microglia reside in the spinal cord and actively participate in the onset, progression and perhaps resolution of post-SCI neuroinflammation. In the current study, we tested the effects of methylene blue on microglia both *in vitro* and in a rat SCI model. We found that methylene blue inhibited the protein levels of IL-1β and IL-18 rather than their mRNA levels in activated microglia. Further investigation indicated that methylene blue deceased the activation of NLRP3 inflammasome and NLRC4 inflammasome in microglia *in vitro*. Moreover, in the rat SCI model, the similar effect of methylene blue on post-SCI microglia was also observed, except that the activation of NLRC4 inflammasome was not seen. The inhibition of microglia NLRP3 inflammasome was associated with down-regulation of intracellular reactive oxygen species (ROS). The administration of methylene blue mitigated the overall post-SCI neuroinflammation, demonstrated by decreased pro-inflammatory cytokine production and leukocyte infiltrates. Consequently, the neuronal apoptosis was partially inhibited and the hind limb locomotor function was ameliorated by methylene blue treatment. Our research highlights the role of methylene blue in inhibiting post-SCI neuroinflammation, and suggests that methylene blue might be used for SCI therapy.

## Introduction

A spinal cord injury (SCI) often starts with an abrupt traumatic impact to the spine which breaks or dislocates the vertebrae. It is a detrimental neurological disease that makes lots of individuals suffer every year. The pathological changes of SCI involve the primary and secondary damage (Silva et al., 2014). Broken bone fragments, displaced disc tissues, ligaments bruise or tear into the spinal cord tissue cause the primary damage when the traumatic injury happens. After that, the primary injury is followed by the cascade of secondary biological changes, which start from minutes and last for weeks post the mechanical injury (Hausmann, 2003; Oyinbo, 2011; Anwar et al., 2016). The secondary changes induce further and extensive neurological injury. Ultimately, the chronic phase takes place days to years post the initial injury, rendering long-term neurological impairments in both orthograde and retrograde directions (Cramer et al., 2005; Yiu and He, 2006).

Neuroinflammation is one of the post-SCI secondary changes. It plays a crucial role in modulating the pathological progression of acute and chronic SCI. It also plays a key role in regulating neuronal damage and regeneration (Beattie, 2004). Microglia reside in the spinal cord and actively participate in the onset, progression and perhaps resolution of post-SCI neuroinflammation (Loane and Byrnes, 2010; Cherry et al., 2014; Zhou et al., 2014). Shortly after SCI, resident microglia are activated and subsequently secret a series of pro-inflammatory cytokines and chemokines to recruit blood-borne leukocytes (Trivedi et al., 2006; Zhou et al., 2014). Due to their critical role in initiating post-SCI neuroinflammation, it would be possible to restrain their inflammatory response so as to curtail the detrimental neuroinflammation.

As an agent in a variety of mitochondria targeted cytotoxicity paradigms, methylene blue is neuroprotective in several experimental neurological disorders (Tsvetkov et al., 2010; Poteet et al., 2012; Shen et al., 2013; Xie et al., 2013). However, the effect of methylene blue on acute SCI has not been elucidated. And the mechanisms of methylene blue-mediated neuroprotection has not been completely revealed. In the current study we tested the influences of methylene blue on microglia both *in vitro* and in a rat SCI model. We found that methylene blue inhibited the protein levels of IL-1β and IL-18 rather than their mRNA levels in *in vitro* activated microglia. Further investigation indicated that methylene blue deceased the activation of NLRP3 inflammasome and NLRC4 inflammasome in microglia. Moreover, in the rat SCI model, the anti-inflammatory effect of methylene blue on post-SCI microglia was observed. The administration of methylene blue mitigated the overall post-SCI neuroinflammation, demonstrated by decreased pro-inflammatory cytokine production and leukocyte infiltrates. Consequently, the neuronal apoptosis was partially inhibited and the hind limb locomotor function was improved by methylene blue treatment. Our research suggests that methylene blue might be applied for SCI therapy.

## Materials and Methods

### Rat SCI Model

The animal study was approved by the Animal Care and Use Committee of Fujian Medical University. All animal experiments were performed in accordance with institutional guidelines of laboratory animals in neuroscience and behavioral research. Male Sprague-Dawley rats (10-week old, 250–300 g) were purchased from Fujian Medical University Animal Center. Rats were anesthetized via inhaling 3% of isoflurane at the flow rate of 1 L/min. Midline skin incisions were made and the T12 spinous processes were exposed. A laminectomy was performed at T12. The compression was applied by placing the base of a compression platform (area 2 × 5 mm^2^) onto the exposed cord. A weight of 50 g was applied steadily to the platform for exact 5 min. After that, the platform was removed, and the muscles and skins were sutured. On each sham-operated rat, a laminectomy was done without compression.

### Methylene Blue Injection

Methylene blue (Sigma-Aldrich) was prepared in sterile PBS. Fifteen minutes before SCI, each rat received an i.v. tail injection of 500 μl of PBS or methylene blue (4 mg/kg body weight). Three hours after SCI, each rat received another i.v. injection of methylene blue at the same dose. To test the effect of methylene blue at a lower dosage, each rat received methylene blue of 2 mg/kg body weight in the same way as above.

### Enrichment of Immune Cells from Rat Spinal Cords

Rats were anesthetized by inhalation of 3% of isoflurane followed by transcardial perfusion with 200 ml of phosphate buffered saline (PBS). The spinal cord was taken, minced into 1 mm^3^ pieces, and treated with RPMI1640 supplemented with 2 mg/ml collagenase IV (Thermo Fisher Scientific), 200 U/ml DNase I (Sigma-Aldrich), 20% fetal bovine serum (FBS) and 2.5 mM CaCl_2_ for 30 min on an orbital shaker while shaking at 150 rpm in a 37°C incubator. Digested tissues were then filtered through 70-μm cell strainers and were overlaid onto 20% Percoll (GE Healthcare) before centrifugation at 250 *g* for 10 min. The cell pellet was resuspended in PBS or culture medium before further treatment.

### Flow Cytometry

To detect and sort indicated immune cells in the spinal cords, the following anti-rat antibodies were used: APC anti-CD3 (IF4), PE/Cy7 anti-CD45 (OX-1), APC anti-CD11b/c (OX-42), PE anti-CD80 (3H5), PE anti-CD86 (24F) and FITC anti-RTIB (MHC-II, OX-6) were bought from Biolegend; FITC anti-CD163 (ED2) was purchased from Bio-Rad; Biotinylated anti-granulocyte (HIS48) was purchased from eBioscience. Cells were stained with 2–5 μg/ml of each antibody on ice for 15 min, and were then loaded onto a BD LSR-II flow cytometer for analysis. Dead cells were excluded by with propidium iodide (2 μg/ml) staining (eBioscience). For cell sorting, cells were sorted on a BD FACSAria II sorter (BD Biosciences). The specificity of antibody staining was shown in Supplementary Figure S1.

### Cell Culture

Sorted microglia were cultured in RPMI1640 (Thermo Fisher Scientific) supplemented with 1% glutamine (Sigma-Aldrich), 10% FBS (Hyclone), 100 U/ml penicillin and 100 μg/ml streptomycin (both from Sigma-Aldrich). 1 × 10^6^/ml cells were cultured in each well of a 24-well plate (Corning). Methylene blue was added into cell culture at indicated concentrations. Immediately after addition of methylene blue, cells were primed with 10 ng/ml lipopolysaccharide (LPS, Sigma-Aldrich) for 6 h. Cells were then treated with 5 mM adenosine triphosphate (ATP, Sigma-Aldrich) for additional 1 h. Methylene blue was always present in the cell culture during LPS and ATP treatment.

### ELISA

The supernatants of microglia culture were collected and stored at −80°C before tests. ELISA was performed using IL-1β ELISA Kit (Abcam, ab100768,) and IL-18 ELISA Kit (Abcam, ab213909) following the manufacturer’s instructions. For tissue cytokine detection, 6 h after SCI, spinal cord tissue at the site of injury was resuspended in a buffer (10 mM Tris, 0.032 mM sucrose, 0.5 mM EDTA, 2 mM EGTA, 1 mM PMSF, 10 μg/ml leupeptin and 10 μg/ml aprotinin, 7.4 pH; 100 mg tissue per ml of buffer) before homogenization and sonication using a Q55 Sonicator (Qsonica) on ice. The concentrations of indicated cytokines were determined with ELISA kits (Abcam, ab46070 and ab100772), respectively.

### Quantitative RT-PCR (q-RTPCR)

RNA was purified from microglia or spinal cord tissues using the Trizol reagent (Thermo Fisher Scientific). cDNA was synthesized with SuperScript^®^ III First-Strand Synthesis System (Thermo Fisher Scientific). q-RTPCR was conducted using Fast SYBR^®^ Green Master Mix (Thermo Fisher Scientific) on a 7300 q-PCR System (Invitrogen). The amplification protocol is: pre-warming at 50°C for 2 min and 94°C for 10 min, followed by 40 cycles of 30 s at 94°C and 1 min at 62°C. Amplification data was analyzed using 2^−ΔΔCt^ method (Livak and Schmittgen, 2001). The primers were designed using Primer-Blast program on the NCBI website following the design method of the program. Primer sequences are listed in Supplementary Table S1.

### Western Blot

Proteins were extracted from cells or tissues using RIPA buffer (Thermo Fisher Scientific, 89900) with protease inhibitor cocktail (Sigma-Aldrich, S8820). Protein quantification was done using Pierce BCA Protein Assay Kit (Thermo Fisher Scientific, 23225). Thirty microgram total protein was loaded onto 4%–20% Mini-PROTEAN TGX Precast Protein Gels (Bio-Rad, 4561094) for electrophoresis. The following antibodies were used for protein detection: anti-α-Tubulin antibody (#2144, 1:1000) was purchased from Cell Signaling Technology. Anti-ASC antibody (sc-514414, 0.4 μg/ml) and anti-caspase-1 antibody (sc-1218, for pro-caspase-1 and p20, 0.5 μg/ml) antibody were purchased from Santa Cruz Biotechnology. Anti-NLRP3 (ab214185, 1:500), anti-NLRC4 (ab99860, 1:1000) and anti-AIM2 (ab180665, 1:1000) antibodies were purchased from Abcam. Anti-IL-1β (NB600-633, for pro-IL-1β and mature IL-1β, 1:1000) antibody was purchased from Novus Biologicals. Optical density was analyzed on a The ChemiDoc XRS+ system (Bio-Rad). The specificity of antibody staining was shown in Supplementary Figure S2.

### Co-Immunoprecipitation

Cells were lysed on ice for 15 min using Pierce IP Lysis Buffer supplemented (Thermo Fisher Scientific, 87787) with protease inhibitor cocktail (Sigma-Aldrich, S8820). Cell lysates were then centrifuged at 2500 *g* for 5 min at 4°C to remove cell debris. Proteins in the cell lysates were quantified with Pierce BCA Protein Assay Kit (Thermo Fisher Scientific, 23225). Co-IP was conducted using the Dynabeads™ Co-Immunoprecipitation Kit (Thermo Fisher Scientific, 14321D) following the vendor’s instructions. Briefly, 14 μg anti-ASC (Santa Cruz Biotechnology, sc-514414), or anti-NLRP3 (Abcam, ab214185) or anti-NLRC4 (Abcam, ab99860) antibody was coupled to 1.5 mg of Dynabeads, respectively. Mouse IgG (Thermo Fisher Scientific, 31903) and rabbit IgG (Thermo Fisher Scientific, 31235) served as the negative control for ASC antibody and other antibodies, respectively. Five-hundred microgram total proteins were then applied to 1.5 mg antibody-coupled Dynabeads in an Eppendorf tube, incubated 30 min on a rotator at 4°C. The tube was then put on a DynaMag™ magnet (Thermo Fisher Scientific, 12320D) for 1 min, and Dynabeads attaching to the walls were washed with wash buffer. Proteins bound to the Dynabeads were eluted with 30 μl of elution buffer. The elutions were then mixed with 30 μl of 2× Laemmli buffer. Five microliter of 1 M Tris buffer (pH 9.8) were added to the mixture to match the pH of the stacking gel. Samples were boiled for 5 min and loaded onto 4%–20% Mini-PROTEAN TGX Precast Protein Gels (Bio-Rad, 4561094) for electrophoresis.

### H2DCFDA Staining

Microglia were sorted from rat spinal cords using flow cytometry. Sorted cells were incubated in PBS containing 10 μM H2DCFDA (Thermo Fisher Scientific) for 45 min in a 37°C incubator. Cells were then washed twice in PBS and H2DCFDA fluorescence was analyzed with flow cytometry.

### Terminal Deoxynucleotidyl Transferase dUTP Nick End Labeling (TUNEL)

Spinal cords were fixed with 4% paraformaldehyde followed by paraffin embedding. Five-micron thick cross sections were cut. The sections were labeled using DeadEnd™ Fluorometric Terminal deoxynucleotidyl transferase dUTP nick end labeling (TUNEL) System (Promega). Images were taken on a Leica DMIRE2 fluorescent microscope.

### Neurologic Evaluation

BBB locomotor test was performed to evaluate the hindlimb locomotor function at pre-injury and 1, 3, 7, 14 and 21 days post SCI. The hindlimb movements during locomotion were quantified using a scale ranging from 0 to 21. Two observers, who were blinded to the group setting, observed each rat for five consecutive minutes at each time point.

### Statistical Analysis

Most experiments were independently performed two or three times, with 3–10 individual samples in each group. Data was shown as mean ± standard deviation and was analyzed by GraphPad Prism 7.0. Student’s *t* test or one-way analysis of variance (ANOVA) was performed for comparison of mean values among the groups. For neurologic evaluation, repeated-measures ANOVA was used. *p* values <0.05 were considered significant.

## Results

### Methylene Blue Inhibits the Production of Mature IL-1β and IL-18 by Spinal Cord Microglia

**Figure 1:**
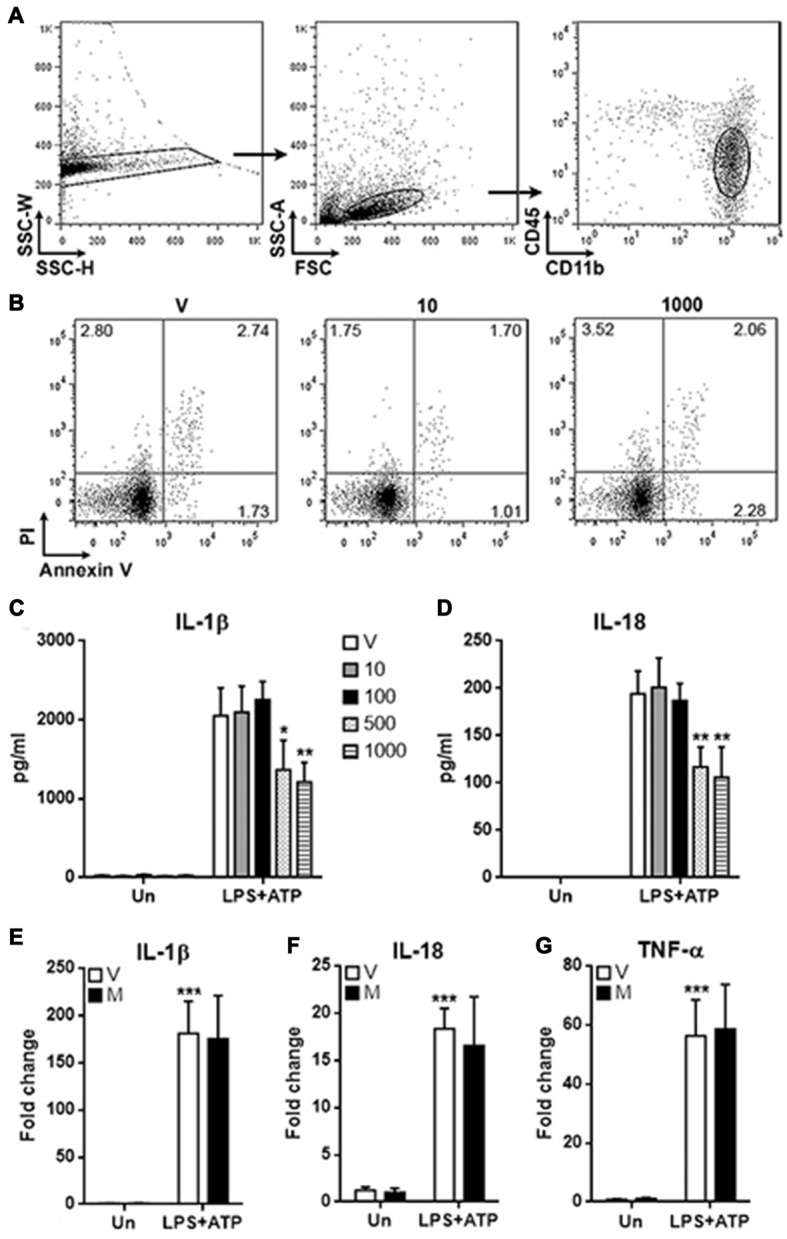
Methylene blue inhibits the production of mature IL-1β and IL-18 by microglia.** (A)** Gating strategy for distinguishing and sorting microglia form normal rat spinal cords. CD45^low^CD11b^+^ cells were microglia. *N* = 3 per group. **(B)** Apoptosis of microglia cultured for 48 h. V: vehicle phosphate bufferedsaline (PBS); 10: 10 nM methylene blue; 1000: 1000 nM methylene blue. Numbers in the quadrats are percentage of each gated population. *N* = 3 per group. **(C,D)** Concentrations of IL-1β and IL-18 in the supernatants. Un: no stimulation. lipopolysaccharide (LPS)+adenosine triphosphate (ATP): stimulation with LPS followed by ATP treatment. V: vehicle; 10~1000: methylene blue concentrations (nM). *p < 0.05; **p < 0.01 in comparison with stimulated cells of the vehicle group. **(E–G)** mRNA levels of IL-1β, IL-18 and TNF-α in cultured microglia. V: vehicle; M: 500 nM methylene blue. ***p < 0.001 in comparison with unstimulated cells of the vehicle group. *N* = 6–8 per group.

To evaluate the potential effects of methylene blue on spinal cord microglia, normal rat spinal cord tissues were taken and digested to prepare immune cell suspensions. As shown in Figure 1A, the CD45^low^CD11b^+^ microglia were distinguished and sorted by flow cytometry. We firstly check the possible toxicity of methylene blue on microglia. Microglia were treated with methylene blue at 10 and 1000 nM for 48 h, and no significant apoptosis was observed (Figure 1B). Microglia were then incubated with LPS and ATP in the presence or absence of methylene blue ranging from 10 and 1000 nM for 6 h, followed by determination of IL-1β and IL-18 concentration in the supernatants. As shown in Figures 1C,D, both IL-1β and IL-18 were decreased by methylene blue at 500 and 1000 nM, and this inhibition was not due to the changes in mRNA levels of these cytokines (Figures 1E,F). The mRNA level of TNF-α was not affected (Figure 1G).

### Methylene Blue Does Not Influence Polarization of Microglia

**Figure 2:**
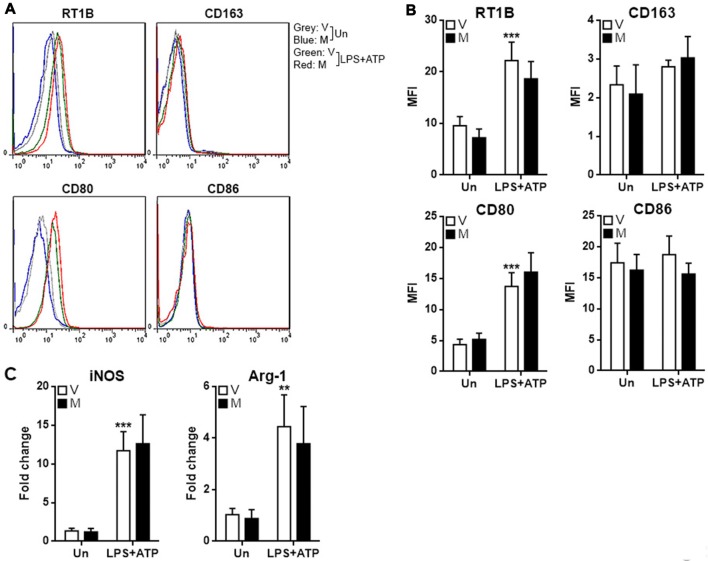
Methylene blue does not alter the expression of M1 and M2 markers.** (A,B)** Expression of indicated surface markers on cultured microglia. Representative flow cytometry histograms are shown in **(A)**, and statistics is shown in **(B)**. **(C)** mRNA levels of iNOS and Arginase-1 in cultured microglia. Un: no stimulation. LPS+ATP: stimulation with LPS followed by ATP treatment. V, vehicle; M, 500 nM methylene blue. **p < 0.01; ***p < 0.001 in comparison with unstimulated cells of the vehicle group. *N* = 4 per group.

To assess if methylene blue impacts M1 and M2 polarization of microglia, M1 and M2 markers were tested in microglia after treatment with LPS/ATP in the presence or absence of methylene blue. LPS/ATP treatment up-regulated the expression of RTIB (rat MHC-II) and CD80, while the expression of CD163 and CD86 were not altered (Figures 2A,B). However, methylene blue did not change the expression of these markers either in resting microglia or stimulated microglia (Figures 2A,B). Moreover, two other M1 and M2 markers, iNOS and Arginase-1, were also up-regulated by LPS plus ATP, but no effect of methylene blue on their expression was observed (Figure 2C).

### Methylene Blue Inhibits Inflammasome Formation in Microglia *in Vitro*

**Figure 3:**
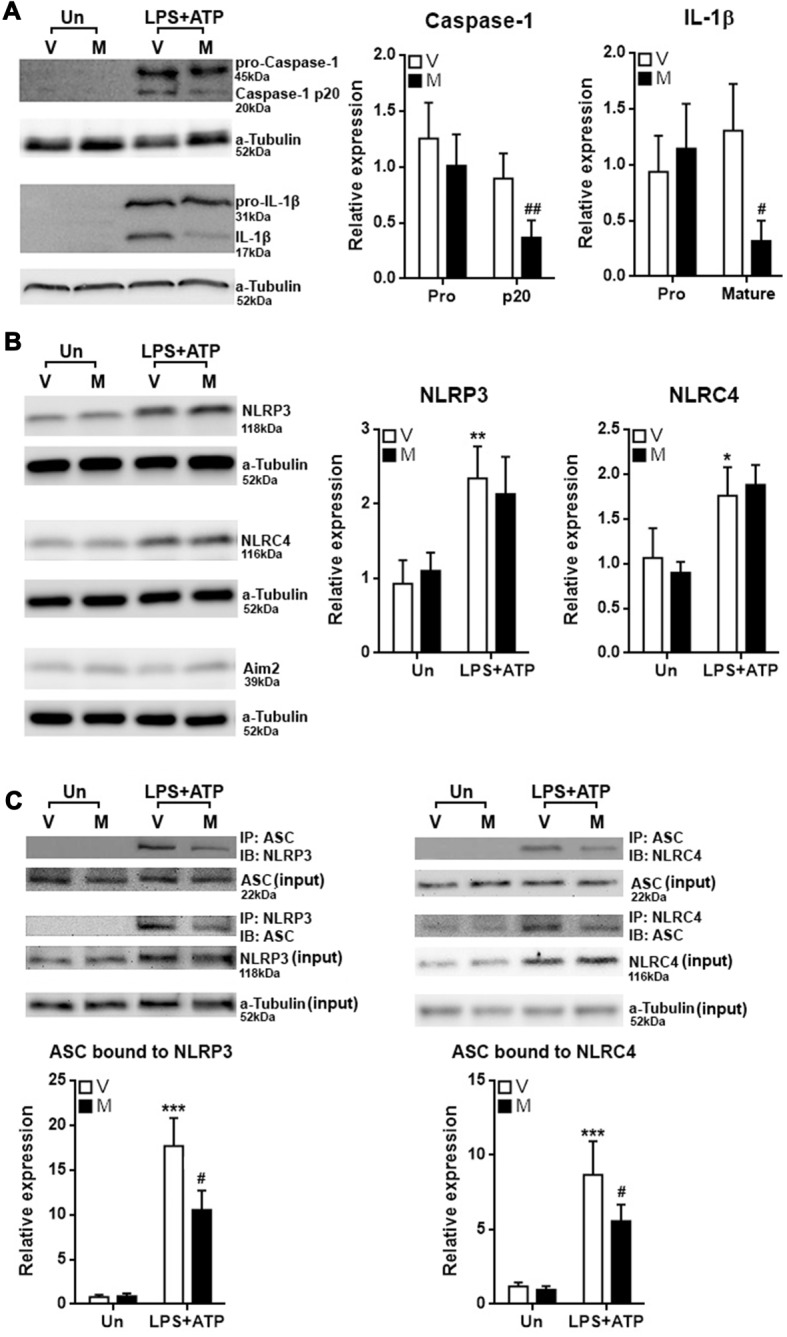
Methylene blue inhibits inflammasome formation in cultured microglia.** (A)** Expression of Pro-Caspase-1, Caspase-1 p20, pro-IL-1β and mature IL-1β in microglia with or without stimulation in the presence or absence of methylene blue. Left: representative Western blot images. Right: statistics. *N* = 5 per group. The **(B)** expression of NLRP3, NLRC4 and Aim2 in microglia. Left: representative Western blot images. Right: statistics. *N* = 4 per group. **(C)** Co-immunoprecipitation assay showing the binding of ASC to NLRP3 or NLRC4. Left: representative Western blot images. Right: statistics. Un: no stimulation. LPS+ATP: stimulation with LPS followed by ATP treatment. V: vehicle; M: 500 nM methylene blue. IP: immunoprecipitation with the antibody against indicated protein. IB: detection of indicated protein. To analyze the relative expression of target proteins, the band intensities of target proteins were normalized to corresponding band intensities of α-Tubulin, followed by calculating the expression in other groups relative to “Un (V)” group. *N* = 4 per group. *p < 0.05; **p < 0.01; ***p < 0.001 in comparison with unstimulated cells of the vehicle group. ^#^*p* < 0.05; ^##^*p* < 0.01 in comparison with stimulated cells of the vehicle group.

The decrease in the production of IL-1β and IL-18 suggested that inflammasome activation might be alleviated by methylene blue, so we checked the cleavage of Caspase-1 and IL-1β in stimulated microglia. As shown in Figure 3A, stimulation with LPS plus ATP substantially increased expression of pro-Caspase-1 and pro-IL-1β, together with up-regulation of cleaved Caspase-1 and IL-1β. Methylene blue reduced the levels of cleaved Caspase-1 and IL-1β under stimulation. We then checked the expression of three important proteins known to participate in inflammasome activation in microglia: NLRP3, NLRC4 and Aim2. In stimulated microglia, the protein levels of NLRP3 and NLRC4 were up-regulated while Aim2 expression was not altered (Figure 3B). We therefore focused on the inflammasome formation mediated by NLRP3 and NLRC4. Co-immunoprecipitation assay demonstrated significant binding of ASC to NLRP3 and NLRC4 after stimulation with LPS plus ATP, suggesting that NLRP3 inflammasome and NLRC4 inflammasome were formed. Methylene blue diminished the binding of ASC to NLRP3 and NLRC4, suggesting that it is an inhibitor of inflammasome activation (Figure 3C; Supplementary Figure S3).

### Methylene Blue Inhibits NLRP3 Inflammasome Formation in Post-SCI Microglia

**Figure 4:**
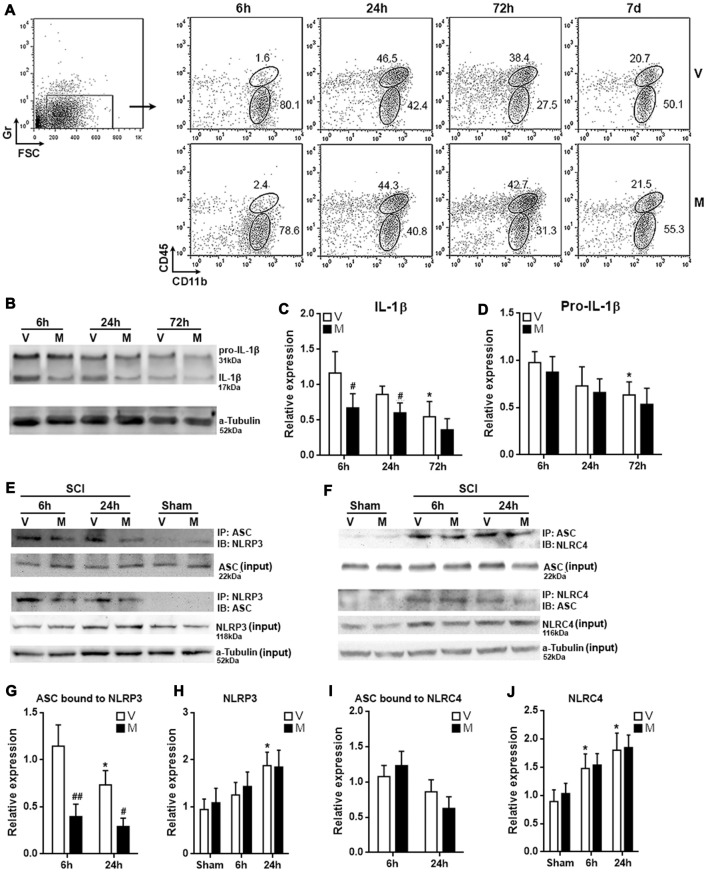
Methylene blue inhibits NLRP3 inflammasome formation in post-spinal cord injury (SCI) microglia.** (A)** Proportions of microglia and macrophages in spinal cords at indicated time points post SCI. Gr^−^CD45^low^CD11b^+^ microglia and Gr^−^CD45^hi^CD11b^+^ macrophages were distinguished in spinal cord immune cells from 6 h to 7 days post SCI. These are representative dot plots of three independent experiments. Note that the leftmost dot plot showing the gating of Gr-negative cells was a sample collected 6 h post SCI. This is why only a small amount of Gr-positive cells were seen. Numbers in the plots are proportions of each gated population. **(B–D)** Expression of pro-IL-1β and mature IL-1β in microglia sorted from post-SCI spinal cords. Representative Western blot images are shown in **(B)**, statistical analysis is shown in **(C,D)**, respectively. *N* = 3 per group. **(E)** Representative images of Co-immunoprecipitation assay for the binding of ASC to NLRP3 in post-SCI microglia. IP: immunoprecipitation with the antibody against indicated protein. IB: detection of indicated protein.** (F)** Representative images of Co-immunoprecipitation assay for the binding of ASC to NLRC4 in post-SCI microglia. **(G–J)** Statistical analysis for the ASC bound to NLRP3 **(G)** or NLRC4 **(I)**, and for the expression of NLRP3 **(H)** or NLRC4 **(J)**. *N* = 5 per group. V: vehicle; M: methylene blue treatment. To analyze the relative expression of target proteins in **(C,D)**, the band intensities of target proteins were normalized to corresponding band intensities of α-Tubulin, followed by calculating the target protein expression in other groups relative to “6 h (V)” group. For the quantification in **(G–J)**, target proteins were normalized to α-Tubulin, followed by calculating the target protein expression in other groups relative to “Sham (V)” group. *p < 0.05 in comparison with the “6 h” vehicle group or sham group (if any). ^#^*p* < 0.05; ^##^*p* < 0.01 in comparison with the corresponding vehicle group at each time point.

The *in vitro* analysis posed the possibility that methylene blue would mitigate neuroinflammation *in vivo*. To test this hypothesis, we administered methylene blue before conducting SCI on rats. Immune cells were then enriched from T12 spinal cord tissues for evaluation of microglia function. In the whole spinal cord immune cell population, Gr^−^CD45^low^CD11b^+^ microglia were distinguished from Gr^−^CD45^hi^CD11b^+^ macrophages (Figure 4A; Supplementary Figure S4). Microglia were sorted by flow cytometry 6 h, 24 h, 72 h and 7 days post SCI, since our preliminary test indicated that expression of mature IL-1β in microglia was up-regulated 6 h post SCI as compared with sham control (Supplementary Figure S5). Mature IL-1β was then gradually decreased from 24 h to 7 days post SCI (Supplementary Figure S5). At each time point, the proportions of microglia were similar between vehicle-treated and methylene blue-treated group, suggesting that methylene blue did not impact microglia proliferation or death after SCI (Figure 4A). The proportions of macrophages were also comparable between the two groups at each time point, suggesting that macrophage recruitment was not remarkably altered by methylene blue (Figure 4A). However, methylene blue lowered the expression of mature IL-1β while not altering pro-IL-1β expression in microglia 6 h and 24 h post SCI (Figures 4B–D). Furthermore, methylene blue reduced the amount of ASC bound to NLRP3 while not influencing the whole NLRP4 expression at these time points, as compared with vehicle group (Figures 4E,G,H; Supplementary Figure S6), suggesting that activation of NLRP3 inflammasome was inhibited by methylene blue. However, the activation of NLRC4 inflammasome was not significantly changed by methylene blue treatment (Figures 4F,I,J; Supplementary Figure S6).

### Methylene Blue Decreases ROS Generation in Post-SCI Microglia

**Figure 5:**
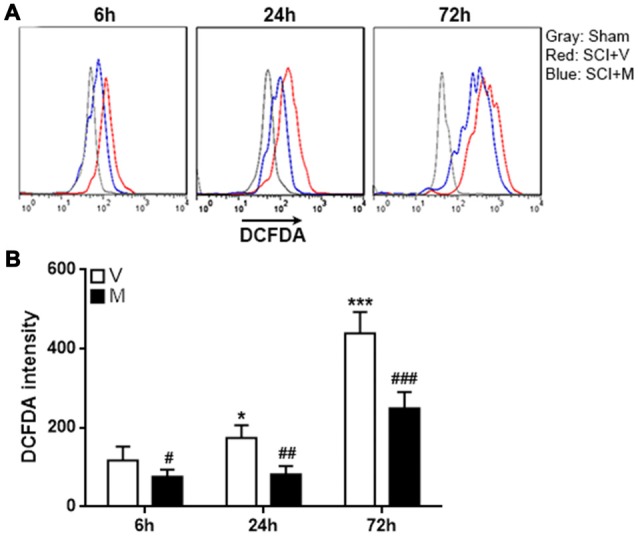
Methylene blue diminished reactive oxygen species (ROS) production in post-SCI microglia.** (A)** Representative histograms indicating the H2DCFDA staining.** (B)** Statistical analysis for the H2DCFDA intensity. V: vehicle; M: methylene blue treatment. *p < 0.05; ***p < 0.001 in comparison with the “6 h” vehicle group. ^#^*p* < 0.05; ^##^*p* < 0.01; ^###^*p* < 0.001 in comparison with the corresponding vehicle group at each time point. *N* = 5 per group.

It has been shown that methylene blue is a potent reactive oxygen species (ROS) inhibitor (Poteet et al., 2012; Xiong et al., 2017), and ROS is important for inducing inflammasome activation in multiple cell types (Zhou et al., 2011; Abais et al., 2015; Jo et al., 2016). To determine whether the effect of methylene blue on inflammasome activation was associated with ROS production, intracellular ROS was analyzed by H2DCFDA staining of microglia. As expected, microglia in methylene blue-treated rats showed lower H2DCFDA intensity, suggesting that methylene blue indeed reduced ROS generation in microglia (Figure 5).

### Methylene Blue Mitigates the Acute Post-SCI Neuroinflammation

**Figure 6:**
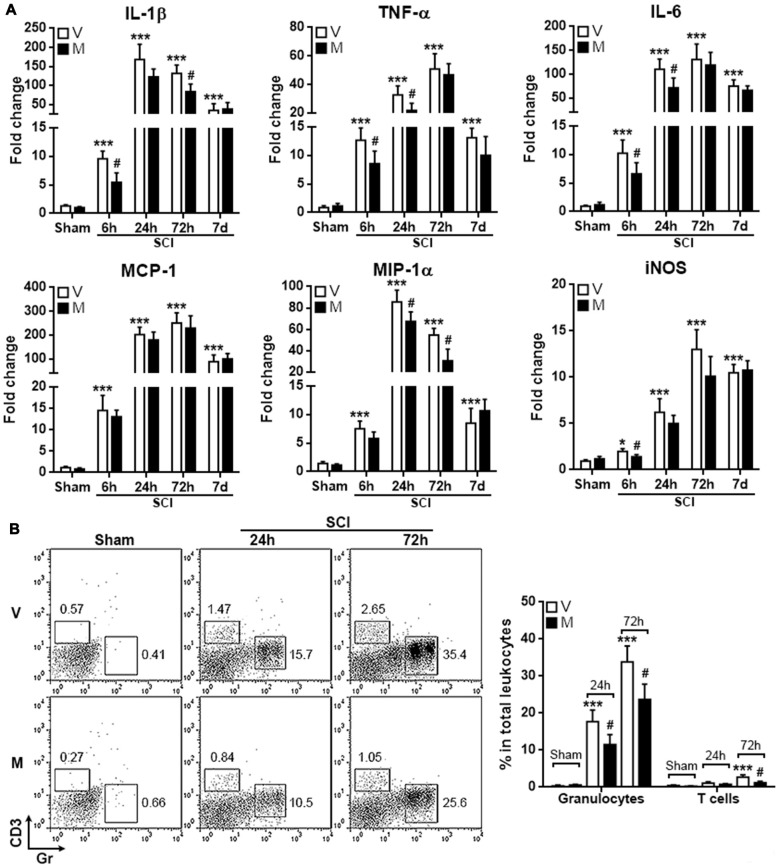
Methylene blue mitigates the acute post-SCI neuroinflammation.** (A)** mRNA levels of indicated molecules at different time points post SCI. *N* = 6 per group. **(B)** Infiltration of CD3^+^ T cells and Gr^+^ granulocytes into injured spinal cords. Left: representative flow cytometry dot plots. Right: statistics. *N* = 5 per group. V: vehicle; M: methylene blue treatment. *p < 0.05; ***p < 0.001 in comparison with sham-vehicle group. ^#^*p* < 0.05 in comparison with the corresponding vehicle group of each time point.

Since microglia are among the first responder cells that initiate neuroinflammation after SCI, the inhibition of inflammasome formation in microglia might mitigate post-SCI neuroinflammation. To test this hypothesis, T12 spinal cord tissues were taken to evaluate the expression pro-inflammatory cytokines and mediators. As shown in Figure 6A, in comparison with vehicle treatment, methylene blue treatment decreased the mRNA level of IL-1β 6 h and 72 h post SCI, while the mRNA levels of TNF-α and IL-6 were moderately decreased 6 h and 24 h post SCI. MCP-1 expression was not altered by methylene blue, whereas MIP-1 expression was down-regulated by methylene blue 24 h and 72 h post SCI. iNOS expression was only reduced in methylene blue-treated rats 6 h post SCI. No significant changes of these molecules were observed in methylene blue-treated rats 7 days post SCI, suggesting that the effect of methylene blue was temporary.

To further confirm the mitigation of post-SCI neuroinflammation by methylene blue, T cell and granulocyte infiltrates in injured spinal cords were quantified. As shown in Figure 6B, SCI induced notable infiltration of CD3^+^ T cells and Gr^+^ granulocytes into spinal cords. Methylene blue treatment reduced the amounts of both T cells and granulocytes, confirming the inhibitory effect of methylene blue on neuroinflammation.

### Methylene Blue Inhibits Cell Apoptosis in Injured Spinal Cords and Improves Function Recovery

**Figure 7:**
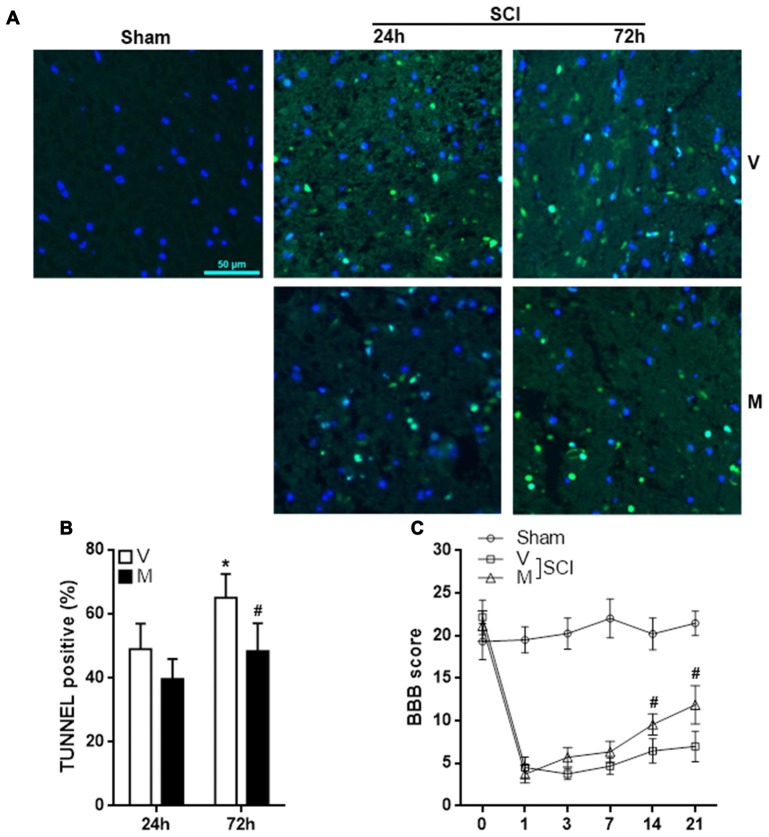
Methylene blue inhibits cell apoptosis in injured spinal cords and improves function recovery.** (A,B)** Terminal deoxynucleotidyl transferase dutp nick end labeling (TUNEL) staining indicating cell apoptosis in spinal cords 24 h and 72 h post SCI. Fluorescent images are shown in** (A)**. Statistics of the proportion of apoptotic cells were shown in** (B)**. *N* = 4 per group.** (C)** BBB score. *N* = 10 per group. V: vehicle; M: methylene blue treatment. *p < 0.05 in comparison with “24 h” vehicle group. ^#^*p* < 0.05 in comparison with the corresponding vehicle group of each time point.

Excessive neuroinflammation causes neuronal death and aggravate tissue damage. To evaluate the effect of methylene blue on post-SCI tissue damage, T12 spinal cord sections were stained with TUNEL kit to assess cell apoptosis. As shown in Figures 7A,B, methylene blue markedly diminished the proportion of apoptotic cells 72 h post-SCI. The apoptotic cells were predominantly neurons (Supplementary Figure S7). To evaluate the hind limb locomotor function post SCI, the BBB score was recorded before SCI and 1, 3, 7, 14 and 21 days post SCI. As shown in Figure 7C, there was no notable difference in the BBB score among sham group, vehicle group and methylene group prior to SCI. SCI induced a sharp reduction in the BBB score in the vehicle group and methylene blue group. A slow and minor increase of the BBB score was seen in the vehicle group, whereas the increase was much quicker and more profound in methylene blue group, suggesting that methylene blue treatment indeed improved hind limb locomotor function post SCI.

**Figure 8:**
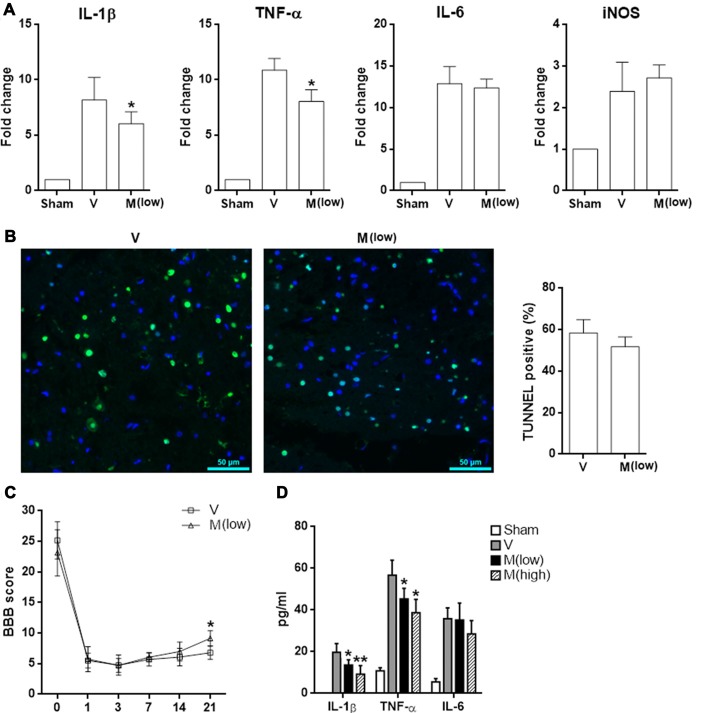
Effect of low dose of methylene blue on SCI. Rats received methylene blue at a lower dosage (2 mg/kg body weight) before and after SCI. **(A)** mRNA levels of indicated pro-inflammatory mediators in T12 spinal cord 6 h after SCI. *N* = 3 per group. **(B)** TUNEL staining indicating cell apoptosis in spinal cords 24 h post SCI. Left panel: fluorescent images. Right panel: statistics of TUNEL-positive cells. *N* = 4 per group. **(C)** BBB score. *N* = 6 per group. **(D)** Concentrations of indicated cytokines in T12 spinal cord lysates 6 h post SCI. *N* = 3 per group. V: vehicle; M (low): methylene blue at low dose; M (high): methylene blue at high dose. *p < 0.05; **p < 0.01 in comparison with vehicle group.

Furthermore, methylene blue at a lower dosage (2 mg/kg body weight) had a weaker anti-inflammatory effect, as demonstrated by less expression of IL-1β and TNF-α in injured spinal cord tissues. However, expression of IL-6 and iNOS has not been affected (Figure 8A). No difference of TUNEL results was observed, suggesting that the low dose of methylene blue could not inhibit neuronal apoptosis (Figure 8B). In terms of limb locomotor function, low dose of methylene blue elicited only a moderate improvement on day 21 post SCI (Figure 8C). ELISA revealed that methylene blue decreased protein levels of IL-1β and TNF-α in the injured spinal cord tissue, while the effect of high dose of methylene blue was more robust (Figure 8D).

## Discussion

In the current study we characterized the effect of methylene blue on the inflammatory response of microglia after SCI. The neuroprotective efficacy of methylene blue has previously been reported in various models (Tsvetkov et al., 2010; Poteet et al., 2012; Shen et al., 2013; Xie et al., 2013). Although some research mentions that methylene blue is able to curtail neuroinflammation in several neurological disorders (Dibaj et al., 2012; Fenn et al., 2015; Xu et al., 2017), the mechanisms of this effect are yet to be elucidated. Importantly, a previous study reports that methylene blue inhibits cytokine and chemokine release from microglia under LPS stimulation, although IL-1β and IL-18 release was not tested in this study (Dibaj et al., 2012). IL-1β and IL-18, which trigger production of secondary pro-inflammatory cytokines and chemokines, are essential in sterile inflammation (Dinarello, 2011; Tripodi et al., 2011). Therefore, the reported anti-inflammatory effect of methylene blue could result from inhibition of IL-1β and IL-18 production. In our *in vitro* experiments, we tested this hypothesis and indeed found that production of IL-1β and IL-18 by LPS-stimulated microglia was decreased by methylene blue. Moreover, the mRNA levels of IL-1β and IL-18 were not influenced, suggesting that the changes took place at the translation or post-translation level.

Maturation of IL-1β and IL-18 in inflammatory cells depends on activation of inflammasomes. The inflammasomes are activated in response to infectious microbes and molecules derived from host proteins. The activated inflammasomes subsequently induce the activation of caspase-1 which cleaves pro-IL-1β and pro-IL-18 into mature IL-1β and IL-18 (Guo et al., 2015; Sharma and Kanneganti, 2016). There are several types of inflammasomes (Latz et al., 2013). In microglia, three types of inflammasomes have been characterized: NLRP3, NLRC4 and Aim2 inflammasomes (Schnaars et al., 2013; Denes et al., 2015; Gustin et al., 2015; Freeman et al., 2017). In our study, we identified up-regulation of NLRP3 and NLRC4 in stimulated microglia. Furthermore, the formation of NLRP3 inflammasome and NLRC4 inflammasome was also observed in stimulated microglia, while methylene blue remarkably reduced the formation of these two inflammasomes. We believe that the inhibition of activation of NLRP3 inflammasome and NLRC4 inflammasome was the cause of decreased production of mature IL-1β and IL-18, since cleaved Caspase-1 was also decreased in methylene blue-treated microglia after stimulation. However, we cannot exclude the possibility that other inflammasomes, such as NLRP1 inflammasome and Aim2 inflammasome were affected by methylene blue. Further study is needed to address this question.

In addition, we also showed that methylene blue had the similar effect on microglia after SCI, although NLRC4 inflammasome activation seemed not inhibited. However, we need to keep in mind that post-SCI microglia might not be completely equivalent to the original microglia before SCI. In our study we used CD45 and CD11b to distinguish microglia and blood-derived infiltrating macrophages. It was possible that some macrophages acquired the microglia phenotype and were indistinguishable from microglia. We thought that the data of 6 h post SCI was the most convincing one because very few circulating macrophages were recruited at this time point. In addition, although we did not test macrophages for inflammasome activation, we guess there might be similar changes to those we saw in microglia, since macrophages and microglia have some common features and functions especially in inflammatory response (Schwartz, 2003; Perry and Teeling, 2013).

Recently, methylene blue was shown to inhibit inflammasome activation (Ahn et al., 2017). Inflammasome activation is a complex process involving different signal pathways, inflammasome components and cofactors (Guo et al., 2015). In particular, ROS especially those from mitochondria are important positive regulators and priming factors of NLRP3 inflammasome activation (Heid et al., 2013; Jo et al., 2016; Minutoli et al., 2016). ROS prime NLRP3 inflammasome via facilitating Ca^2+^ influx, transcriptional regulation of *NLRP3*, and deubiquitylation of NLRP3 (Latz et al., 2013). Therefore, ROS positively regulate microglia activation in various neuropathological conditions, including peripheral nerve injury-induced neuropathic pain and SCI (Block et al., 2007; Kim et al., 2010; Choi et al., 2012). Interestingly, methylene blue is a potent ROS inhibitor which decreases ROS quantity in multiple cell types (Riedel et al., 2003; Poteet et al., 2012; Duicu et al., 2017). In our study, we did observe decrease of ROS in microglia using H2DCFDA staining, suggesting that the methylene blue-induced inhibition of ROS generation might be related to inhibition of NLRP3 inflammasome activation. However, as ROS are involved in several signaling pathways, defining the exact signaling pathway that methylene blue functions on remains challenging and needs further investigation.

The *in vivo* study confirmed the neuroprotection and down-regulation of overall neuroinflammation by methylene blue treatment. However, the impact of methylene blue on microglia response might be just one aspect of the whole neuroprotective effect. Methylene blue could also functions on neurons, astrocytes, vascular endothelial cells and neural stem cells directly or indirectly. Dissection of methylene blue’s efficacy on each cell type shall be done in the future. Additionally, we just focused on the acute neuroinflammation within 3 days post SCI. Whether methylene blue favors tissue repair and neuron regeneration also needs further research.

In conclusion, we found that methylene blue inhibited the inflammatory response of microglia after SCI through alleviation of NLRP3 inflammasome activation. Our research suggests that methylene blue might be applied for SCI therapy.

## Author Contributions

Z-HL, S-YW, L-LC and W-PL were involved in the design of the study, carried out the experiments and participated in the data analysis and manuscript preparation. J-YZ, Q-FK and D-RX contributed to the data analysis and interpretation of the results. All authors read and approved the final manuscript.

## Conflict of Interest Statement

The authors declare that the research was conducted in the absence of any commercial or financial relationships that could be construed as a potential conflict of interest. The reviewer SV and handling Editor declared their shared affiliation.
