# Supplementary material for: Methylene Blue Mitigates Acute Neuroinflammation after Spinal Cord Injury through Inhibiting NLRP3 Inflammasome Activation in Microglia
Source: Front Cell Neurosci. 2017 Dec 11;11:391. doi: 10.3389/fncel.2017.00391 (PMC5732444; doi:10.3389/fncel.2017.00391)
Supplement: Supplementary file 1 [file Data_Sheet_1.docx]

**Supplementary table S1. Primer sequences for real-time PCR**

| **Gene** | **Forward (5’ to 3’)** | **Reverse (5’ to 3’)** |
| --- | --- | --- |
| β-actin | ACAACCTTCTTGCAGCTCCTC | CTGACCCATACCCACCATCAC |
| IL-1β | TGTCTGACCCATGTGAGCTG | GCCACAGGGATTTTGTCGTT |
| TNF-α | TCGGTCCCAACAAGGAGGAG | GGGCTTGTCACTCGAGTTTTG |
| IL-6 | ACTTCACAAGTCGGAGGCTT | TTCTGACAGTGCATCATCGCT |
| CCL2 | AGCATCCACGTGCTGTCTC | TCTTGTAGTTCTCCAGCCGAC |
| CCL3 | TCTGTTACCTGCTCAGCACC | TCGTGGAATTTGCCGTCCATA |
| iNOS | TGGTGAGGGGACTGGACTTT | ACCAACTCTGCTGTTCTCCG |
| Arg1 | GGACATCGTGTACATCGGCT | CTTCCTTCCCAGCAGGTAGC |





**Supplementary Figure S1**. **Staining effects of indicated flow cytometry antibodies.** Rat splenocytes were used for the detection. Note that in normal splenocytes, CD163 staining is weak. Isotype: isotype control. Antibody: antibodies against each protein, respectively.


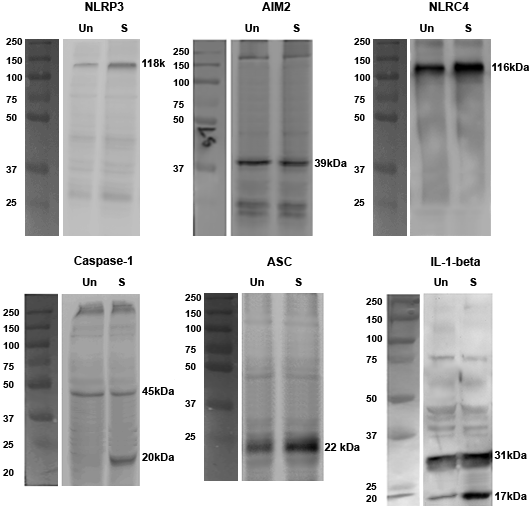


**Supplementary Figure S2**. **Specificity of antibodies against indicated inflammasome components.** 1 × 10^6^/ml rat peritoneal macrophages were primed with 10 ng/ml lipopolysaccharide (LPS) for 6 h followed by treatment with 5 mM adenosine triphosphate (ATP) for additional 1 h. Cells were then lysed for Western blot assay. Un: unstimulated cells. S: cells stimulated with LPS and ATP.


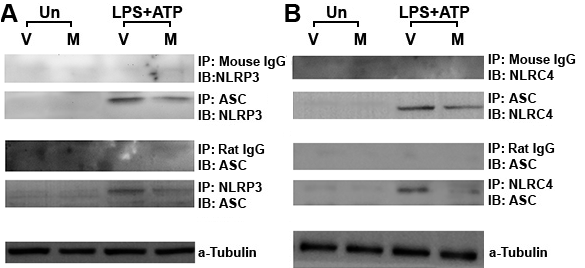


**Supplementary Figure S3**. **Negative control of Co-IP for cells.** Rat peritoneal macrophages were primed with 10 ng/ml lipopolysaccharide (LPS) for 6 h followed by treatment with 5 mM adenosine triphosphate (ATP) for additional 1 h. Rabbit IgG (negative control for NLRP3 and NLRC4 antibodies. Thermo Fisher Scientific), mouse IgG (negative control for ASC antibody. Thermo Fisher Scientific), or indicated antibodies were used for Co-IP assay. The α-tubulin bands show the total protein input for each sample. **(A)** Negative control for IP with NLRP3 and ASC antibodies. **(B)** Negative control for IP with NLRC4 and ASC antibodies. Un: unstimulated cells. LPS+ATP: cells stimulated with LPS and ATP. V: vehicle; M: 500 nM methylene blue. IP: immunoprecipitation with the antibody against indicated protein. IB: detection of indicated protein.


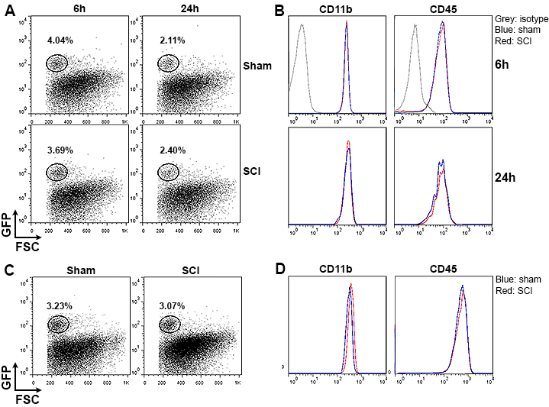


**Supplementary Figure S4**. **Expression of CD45 and CD11b on transferred microglia and macrophages indicates that macrophages and microglia did not convert to each other.** GFP-transgenic 10-week old Sprague-Dawley rats were purchased from Cyagen Biotechnology. With flow cytometry sorting, F4/80^+^ macrophages were enriched from rat spleens, F4/80^+^ microglia were enriched from rat spinal cords (in normal spinal cords, macrophages are rare. So F4/80^+^ spinal cord cells are predominantly microglia.) 1 × 10^4^ GFP^+^ macrophages or microglia were resuspended in 1 μl of artificial cerebrospinal fluid. Before SCI and when T12 spinal cord was exposed, intraparenchymal injections of enriched cells were conducted into T12 using 33 gauge needles and 100-μl NanoFil syringes. Each injection took about 30 seconds followed by a 30-second pause before slowly withdrawing the needle. The center of the injection was targeted intermediate of the dorsal horn and close to the lateral funiculus. After the injection, SCI was induced in each rat. Six hours or 24 hours after SCI, the T12 spinal cord was collected and pressed through 45-μm cell strainers to make single cell suspensions. Cells were then stained with anti-CD45 and anti-CD11b antibodies as described in “Materials and methods”. The expression of CD45 and CD11b on GFP^+^ cells was analyzed. **(A)** GFP^+^ microglia in spinal cords. **(B)** Histograms of CD45 or CD11b staining on GFP^+^ microglia 6 hours or 24 hours after SCI. **(C)** GFP^+^ macrophages in spinal cords 24 hours after SCI. **(D)** Histograms of CD45 or CD11b staining on GFP^+^ macrophages 24 hours after SCI. This is a representative of two independent experiments.


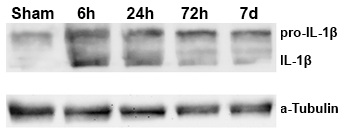


**Supplementary Figure S5**. Expression of IL-1β in microglia 6 hours, 24 hours, 72 hours and 7 days after SCI. This is a representative image of two independent experiments.


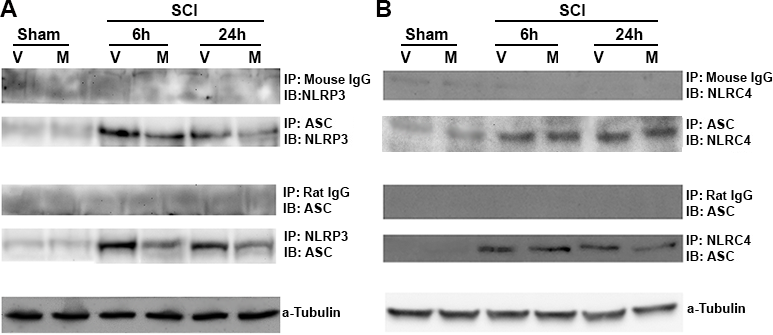


**Supplementary Figure S6**. **Negative control of Co-IP for spinal cord lysates.** Rabbit IgG (negative control for NLRP3 and NLRC4 antibodies. Thermo Fisher Scientific), mouse IgG (negative control for ASC antibody. Thermo Fisher Scientific), or indicated antibodies were used for Co-IP assay. The α-tubulin bands show the total protein input for each sample. **(A)** Negative control for IP with NLRP3 and ASC antibodies. **(B)** Negative control for IP with NLRC4 and ASC antibodies.


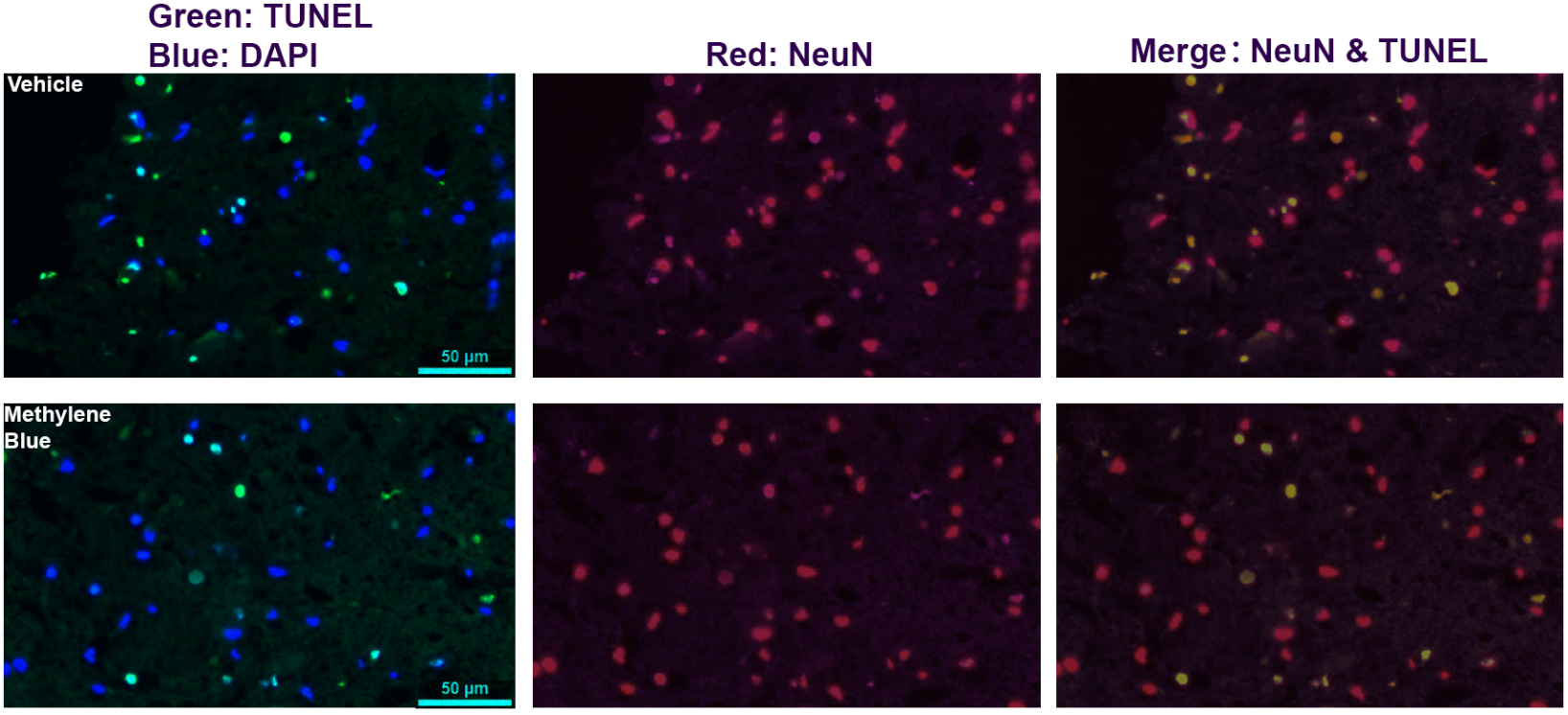


**Supplemental figure 7**. Staining of TUNEL plus NeuN in spinal cord tissue 72 hours post SCI. TUNEL staining was conducted the same way as described in the “Materials and Methods”. In addition, during the labeling step of TUNEL (incubation with TdT reaction mix at 37 ^o^C for 1 hour), 1 μg/ml mouse anti-NeuN monoclonal antibody (Abcam, ab104225) was added together with TdT reaction mix and incubated at 37 ^o^C for 1 hour. The section was then washed and incubated with 2 μg/ml Alexa Fluor^®^ 594 Goat anti-Rabbit IgG at room temperature for 30 min. Vehicle: vehicle control. Methylene blue: high dose of methylene blue.
